# Reading as functional coordination: not recycling but a novel synthesis

**DOI:** 10.3389/fpsyg.2014.01046

**Published:** 2014-09-26

**Authors:** Thomas Lachmann, Cees van Leeuwen

**Affiliations:** ^1^Cognitive and Developmental Psychology Unit, Center for Cognitive Science, University of KaiserslauternKaiserslautern, Germany; ^2^Experimental Psychology Unit and Laboratory for Perceptual Dynamics – University of LeuvenLeuven, Belgium

**Keywords:** reading acquisition, visual processing, analytic vs. holistic processing, literacy, developmental dyslexia, congruence effect, child development

## Abstract

The Functional Coordination approach describes the processes involved in learning to read as a form of procedural learning in which pre-existing skills, mainly from the visual, and auditory domain, are (1) recruited, (2) modified, and (3) coordinated to create the procedures for reading text, which form the basis of subsequent (4) automatization. In this context, we discuss evidence relating to the emerging prevalence of analytic processing in letter perception. We argue that the process of learning to read does not have to lead to a loss of perceptual skill as consequence of a “cultural recycling”; learning to read just leads to a novel synthesis of functions, which are coordinated for reading and then automatized as a package over several years. Developmental dyslexia is explained within this framework as a Functional Coordination Deficit (Lachmann, 2002), since the coordination level is assumed to be most liable to manifest deficiencies. This is because, at this level, the greatest degree of fine tuning of complex functions is required. Thus, developmental dyslexia is not seen as a consequence of a deficient automatization per se, but of automatization of abnormally developed functional coordination.

## ARE LETTERS SPECIAL?

Reading is so much part of everyday life that normally we do not realize how complex this skill is, and how arduous it was to acquire. Reading is a secondary process: beginning readers draw on established cognitive and sensory abilities that are recruited, modified, and coordinated in novel ways to establish the specific strategies of information processing that are optimized for text. According to the *neuronal recycling hypothesis* (Dehaene and Cohen, 2007; Dehaene et al., 2010), these processes may even have the consequence that some of original information processing skills are reduced, as original resources are being redeployed for achieving the newly required functionality. Here we will consider to what extent this may apply to one basic component processing skill: that of analytic visual processing.

Letters, which form the smallest meaningful units of a written text, are not any different in their physical characteristics from meaningless small scribbles, signs of a writing system we don’t understand, or simple geometric shapes. That is, prior to learning to read, letters, and non-letters will not be processed in any systematically different ways. However, even prior to learning to read, such simple items are not natural objects. The latter are most likely 3-dimensional, can be seen in different orientations, can move in space over time, and can occur in cluttered environments, in which they often are partially occluded. All these characteristics necessitate that for natural objects, we make the best out of what is visually available. When an object is partially occluded, we may use global object characteristics such as symmetry to complete them perceptually. We make the most out of an object, if we concentrate on its invariant properties, for instance properties that remain unchanged under positional transformations and different orientations, and we are poised to take clues from the context as to what the nature of the object may be.

Even though those small scribbles and simple geometric drawings are not natural objects, it is plausible to assume that they still trigger these processes. For instance, effects of mental rotation were found to be similar for both 2- and 3-dimensial objects (Shepard and Metzler, 1971; Cooper and Shepard, 1973) and visual completion is based on criteria of mergability of 3-dimensional volumes, both in actual 3-dimensional occluded objects, and in 2-dimensional drawings of them (Tse, 1999). In other words, we may observe that there is, even though with individual differences depending on age (Dror et al., 2005) gender (Alexander and Evardone, 2008; Jansen and Kaltner, 2013) and stimulus material (Geiser et al., 2006) a robust over-all tendency to perceive natural objects *holistically*, and that these preferences extend to 2-dimensional drawings.

Yet, also prior to learning to read, natural 3-dimesional objects, and 2-dimensional drawings alike, can already be perceived in another mode as well, i.e., *analytically*. The analytic-holistic distinction is a broad one known under a variety of, often conflicting terminology laden with theoretical baggage. Here we simple mean to address a collection of empirical distinctions, depending on the extent to which a perceptual configuration is perceived as independent of its context, the extent to which the percept emphasizes properties of the parts over the whole, the extent to which it is tolerant with respect to the constraints non-local properties impose on component organization^1^, and the extent to which it is oblivious to transformational invariants and/or symmetries. We speak of analytic, when some or all of this applies, and of holistic if otherwise.

Whereas perception is naturally holistic to various degrees, it is sometimes efficient to use an analytic strategy. Consider that while holistic perception would not allow us to see the tiger hiding in the bushes, analytic perception may be able to beat the camouflage. When finding an object, or a path, is difficult, we shift from holistic to analytical strategies and scan parts of the scene or display serially, one by one, in small fragments. As soon as we start doing so, we automatically become oblivious to global symmetries of objects that normally play an implicit role in their identification (Hogeboom and van Leeuwen, 1997; Roelfsema and Houtkamp, 2011; Korjoukov et al., 2012).

## LEARNING TO READ

**FIGURE 1:**
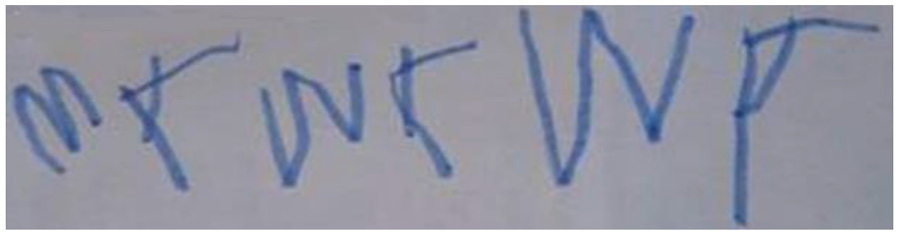
**Children in a very early stage of learning to read do not care about letter orientation, letter order or the fact that single letters represent certain phonemes.** Instead, reading and writing is based on graphic features. Word: “MAMA, Artist: Anton Lachmann (4; 6).

Learning to read involves both holistic and analytic perception, and both are playing different roles during the development of several reading and writing-related sub-skills. According to Frith (1985; see also Chall, 1983; Ehri, 1995), at the beginning of the process of learning to read, *logographic skills* prevail (logographic phase); in this phase, letter configurations will be perceived, just like non-letter ones, in an orientation-unspecific way (see **Figure 1**). The order of letters in a word and other phonological factors are more or less ignored. Unfamiliar words and non-words cannot be read. In fact, instead of “read” we should better use the term “recognized,” because in this stage, the child recognizes a word as a whole and reproduces (“writes”) it as such, mainly based on salient graphic features, just as in object recognition.

Strictly speaking, the logographic sub-skills do not qualify as “reading” or “writing.” This requires the knowledge and use of individual graphemes and phonemes and their correspondences. If this knowledge is available for use, the *alphabetic sub-skill* is developed (alphabetic phase, Frith, 1985, 1986). This sub-skill involves analytic processing; the letters of a word, i.e., the graphemes, are decoded into the corresponding sound one by one, and the sounds are merged together into syllables and words. Fine details of each individual grapheme, its orientation and the order of the graphemes in the configuration are crucial in this stage. Known words, unknown words, as well as non-words can be pronounced, quite likely correctly, i.e., if the correspondence between grapheme and phoneme for the word is according to the learned rule (as for regular words and most words of transparent orthographies, e.g., Italian). In this phase of learning to read, analytic processing is essential. First of all, this is because initially, identifying letters in the context of written text is difficult, and in this case an analytic strategy may be useful. Second, orientation-invariance is not helpful to identify letters; clearly, a “b” is not a “d” nor a “p” nor a “q” either, but also more generally the identity of letters depends on their orientation (van Leeuwen and Lachmann, 2004). Third, and most importantly: the analytic strategies helps establishing a connection with phonology. In skilled readers letters are represented for cross-modal usage (Froyen et al., 2008; Blau et al., 2010; Blomert, 2011), not as a purely visual item, but as connected with auditory information.

More important to reading than auditory categorization are the phonological categories (“a listener will identify as a /*b*/ quite a large number of acoustically different sounds,” Liberman et al., 1957, p. 358, e.g., when spoken by a man or by a woman) developed in this phase of reading acquisition. But just like letters are not natural objects of visual perception, phonemes are not natural objects of auditory perception. The system of phonemic representation gains prominence in the process of learning to read, evolving along with the graphemic representation (Serniclaes et al., 2005; Port, 2007). In transparent languages, such as Italian, the grapheme-phoneme mapping is almost 1:1, but even in the most intransparent cases, morphological units below the word level can be informative with respect to the phonetic expression. This means that in a representational system optimized for efficiency of reading and writing, the building blocks of linguistic codes will emerge that take the form of cross-modal, visual-acoustic (grapheme-phoneme) units (Froyen et al., 2008; Blomert, 2011).

As a consequence of reading expertise in the orthographic phase (Frith, 1985, 1986) of reading acquisition, a sub-skill is developed which enables the instant analysis of larger grapheme units into orthographic units which ideally coincide with morphemes. As a consequence, words can be read as a whole, i.e., without a one-by-one grapheme-phoneme conversion. In this level of processing, the holistic mode again dominates (Wong et al., 2011). Note, however, that this observation is perfectly compatible with the cross-modal character of the representation.

Even though the holistic orthographic sub-skill is relatively effortlessly applied in reading, even in expert readers the analytic alphabetic sub-skill may still be running in parallel (Van Orden et al., 1990) or, at least, remain available for unfamiliar or foreign words (Morton, 1969; Coltheart, 1978, 2007; Davelaar et al., 1978) for both transparent and non-transparent orthographies (Lachmann et al., 2010). Thus, the analytic processing skill remains important even after learning to read has fully been established.

## ANALYTIC PROCESSING OF LETTERS IN EXPERIMENTAL STUDIES

We may conclude that analytic processing is likely to be more specifically associated with reading letters as compared with processing similar non-letter objects. We tested this prediction in a variety of experimental tasks involving different aspects of analytic processing, three of which we will describe in some more detail in the following sections.

**FIGURE 2:**
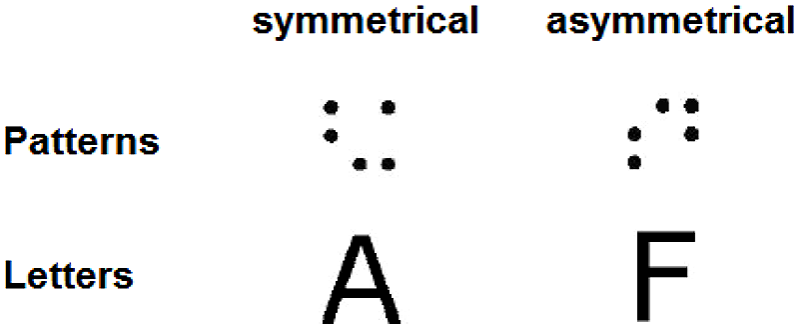
**Examples of symmetrical and asymmetrical dot-pattern (first used by Garner and Clement, 1963) and letter stimuli used in Lachmann and van Leeuwen (2007)**.

One set of experiments deals with the perception of symmetry (Lachmann and van Leeuwen, 2007). Letters and dot patterns (five-dot patterns as first used by Garner and Clement, 1963), with different degrees of symmetry, were presented in a *same–different* task (see **Figure 2**). It had previously been established that the symmetry of the dot patterns is decisive for the speed and accuracy of their comparison (Lachmann and Geissler, 2002; Hermens et al., 2013; Takahashi et al., 2013): symmetrical dot patterns are processed faster (depending in an almost perfectly predictable way on the number of symmetries or, according to Garner and Clement, 1963, the pattern *Goodness*). It is safely to assume, therefore, that these patterns are processed holistically. If letters are processed in a similar way, we should observe symmetry advantages for letters as well. However, in normal reading school-children of the study by Lachmann and van Leeuwen (2007), symmetry effects were observed for dot patterns but not for letters. Interestingly, in this study, age-matched children diagnosed with developmental dyslexia showed the symmetry advantage for both patterns and letters. In addition, this group of children showed transfer between letter and non-letter stimulus blocks, whereas normal reading children did not. The remarkable consequence is that dyslexics are *faster* on this task, in particular also with letters, than normal readers. We interpret this seemingly paradox result (i.e., that developmental dyslexics performed better then controls in a letter task) as indicating that normal readers differentiate in their perceptual strategy between letters and non-letter shapes, whereas dyslexics do not. For the particular task in described study (letters of different orientation have to be rated as “same”), this led to a processing advantage for the latter group. Since analytic and holistic strategies both are available to the normal readers, why then is it the case that for this task the normally reading control children did not apply the holistic strategy to letters too, since this seems to work best for the given task? One possibility is because these readers have automatized the analytic strategy for letters.

Does our result mean that, as recent adoptions of the cerebellar theory (Fawcett, 2002) suggests, developmental dyslexics have a deficit in automatization (Nicolson and Fawcett, 2011)? A deficit in automatization may indeed result in dyslexics failing to automatically apply analytic processing to letters, which happens to be of advantage for the particular version of the *same–different* task used in Lachmann and van Leeuwen (2007), which involved responding to rotated/mirror-imaged versions of two items as “same.”

**FIGURE 3:**
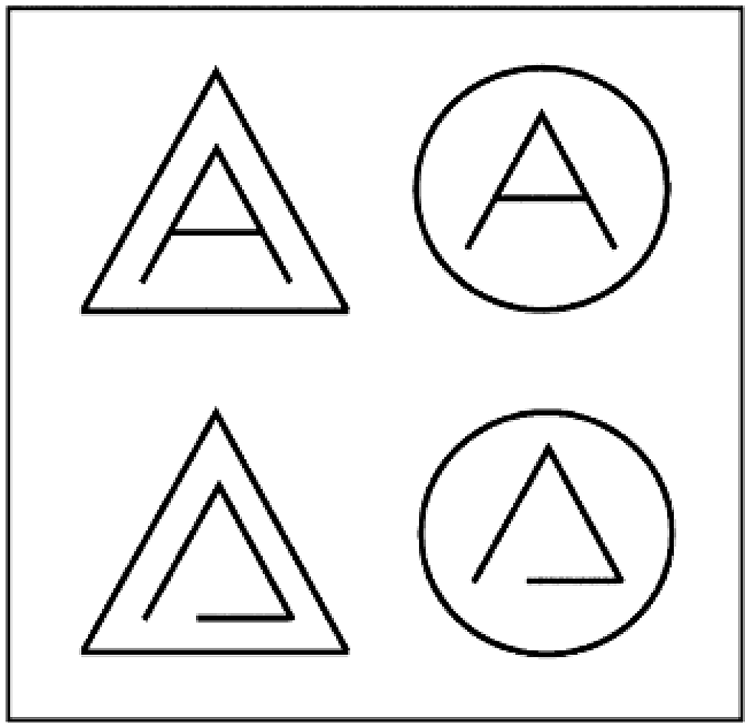
**Letters **(top)** and pseudo-letters **(bottom)** in congruent **(left)** and incongruent **(right)** surroundings, as used in our flanker studies.** See also Fernandes et al. (2014) for similar stimuli.

But the automatization deficit approach cannot explain a number of effects (Rusiak et al., 2007), as for instance the ones observed in another set of experiments using stimuli such as those displayed in **Figure 3** (Lachmann and van Leeuwen, 2004, 2008b; van Leeuwen and Lachmann, 2004; see also Fernandes et al., 2014). Similarly to Eriksson’s classical Flanker study (Eriksen and Eriksen, 1974), we investigated effects of congruence of the surrounding context on the processing of the central target. Non-pseudo- and rotated letter targets all show positive effects of flanker congruence, i.e., processing is facilitated if the surroundings are similar in shape to the central target. According to our terminology, this implies that these items are processed holistically. Interestingly, for letters the surrounding shape congruency is irrelevant^2^, which is reflected in absence of congruence effects, or even interferes with processing, leading to a *negative* congruence effect (Bavelier et al., 2000; Briand, 1994; van Leeuwen and Bakker, 1995). These effects can be explained by assuming that letters are processed analytically; in cases where the surrounding context makes analytic processing difficult the surrounding context is actively suppressed, resulting in negative congruence effects: more effort is needed to suppress a congruent than an incongruent context.

Variations of this paradigm have been informative about the strategic character of the processing dissociation between letters and non-letter shapes. First, the dissociation is task-dependent. *Positive* congruence effects in letters appear in conditions where the task can be performed by identifying the global shape of the items (Lachmann and van Leeuwen, 2004; van Leeuwen and Lachmann, 2004). This means that the holistic processing strategy for letters is still available and is likely to be recruited if it is recognized to be beneficial to the task. Second, the process dissociation between letters and non-letter shapes has been studied in developmental dyslexics and was compared to that of normally reading controls (Lachmann and van Leeuwen, 2008a; Fernandes et al., 2014). Fernandes et al. (2014) replicated the aforementioned dissociation between letters and non-letters in normal readers, but found that it is absent in developmental dyslexics (depending on their phonological recoding skills). In other words, dyslexics in this study failed to apply the analytic strategy – in line with our results from the symmetry paradigm. Interestingly, a seemingly contrasting result for dyslexics was obtained in Lachmann and van Leeuwen (2008a); here, the largest subgroup of developmental dyslexics showed a *negative* congruence effect, much more strongly than the normal readers. Besides methodological differences (e.g., shorter presentation rate, different stimuli, and different diagnostic criteria), between the two studies, this discrepancy can also be explained on the basis of the specific context from which the dyslexics in the latter study were recruited: in our study they were pupils of a special concentration school, which provided intensive training to its dyslexic pupils. The training strongly emphasizes the grapheme-phoneme correspondence. In other words, for these dyslexics, unlike those in the Fernandes et al. (2014) study, who did not receive this intensive and specific kind of training, their background strongly encouraged them to use an analytic strategy (as in the alphabetic phase at the beginning of the process of learning to read), even though they must have found this hard. Given that doing so is difficult for them, this can explain that they showed a negative congruence effect. Thus, overall, the results of both dyslexia studies are in good mutual agreement.

**FIGURE 4:**
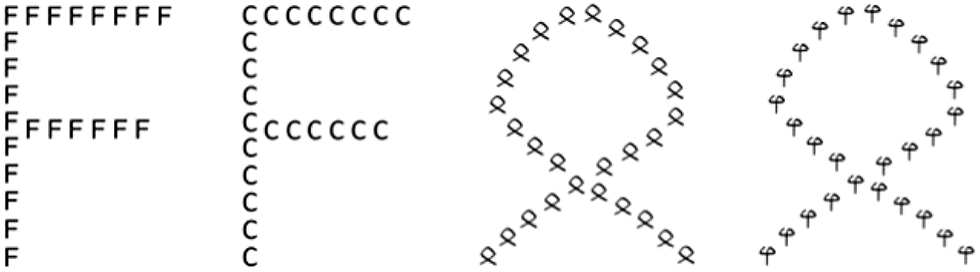
**Illustration of the hierarchical stimuli presented in a study by Lachmann et al. (2014) using the Navon paradigm (Kinchla, 1974; Navon, 1977).** Left side: examples for letters, right side: examples for non-letters. First stimulus example: the local and the global level consists of the same letter F (congruent letter stimulus). Second stimulus example: the global-level letter (F) differs from the local level one (C). Third stimulus example: congruent non-letter stimulus; fourth stimulus example: incongruent non-letter stimulus.

A third experimental method which we used in order to study analytic processing in letters is found in Lachmann et al. (2014, current research topic). This study used the well-known Navon paradigm (Kinchla, 1974; Navon, 1977; see Kimchi, 2014, for a review). The Navon paradigm typically uses compound letters, e.g., a large F composed of a number of identical small Fs or a large H composed of small Hs (congruent), or a large F composed of small Hs or a large H composed of small Fs (incongruent; see **Figure 4**). The large letters are called “global” items, the small ones “local” items. The instruction is varied in a way that a response has to be given either to the local or to the global level, while ignoring information provided in the other level, respectively. With this type of stimuli, global precedence has been established, i.e., faster processing of the global level than the local level (global advantage effect), and an asymmetric congruence effect: incongruency interferes with the local-level target responses but not with global level ones. We may consider both these effects combined as reflecting holistic processing. Thus, the global precedence effect might seem to be in contrast to what one would expect, intuitively, if letters are preferably processed analytically. Note, however, that the global precedence effect strongly depends on the presentation mode (see Kimchi, 2014 for a review) and that the viewing conditions in which the effect is typically found do not resemble those of our flanker/symmetry studies. In Lachmann et al. (2014) we therefore used conditions for which analytic letter processing is expected, because the size and foveal presentation more closely resemble conditions of fluent reading, so the automatized reading specific visual processing strategy was more likely to kick in. With the global stimulus size close to the functional visual field in word reading and local stimuli close to the critical size for fluent reading of individual letters, we compared the global precedence effect for letters and non-letters in central viewing. With these conditions we found the global precedence effect to remain robust for non-letters. For letters, in contrast, the effect disappeared. We interpret these results as according to the view that reading is based on analytic visual processing strategies for letters. In other words, the dissociation in analytic and holistic processing between letters and non-letter shapes is manifest also in the Navon-paradigm, but is limited to viewing conditions that are akin to reading. The automatization of analytic processing for letters, therefore, is highly context-specific.

## READING AS PROCEDURAL LEARNING: AUTOMATIZATION OF FUNCTIONAL COORDINATION

**FIGURE 5:**
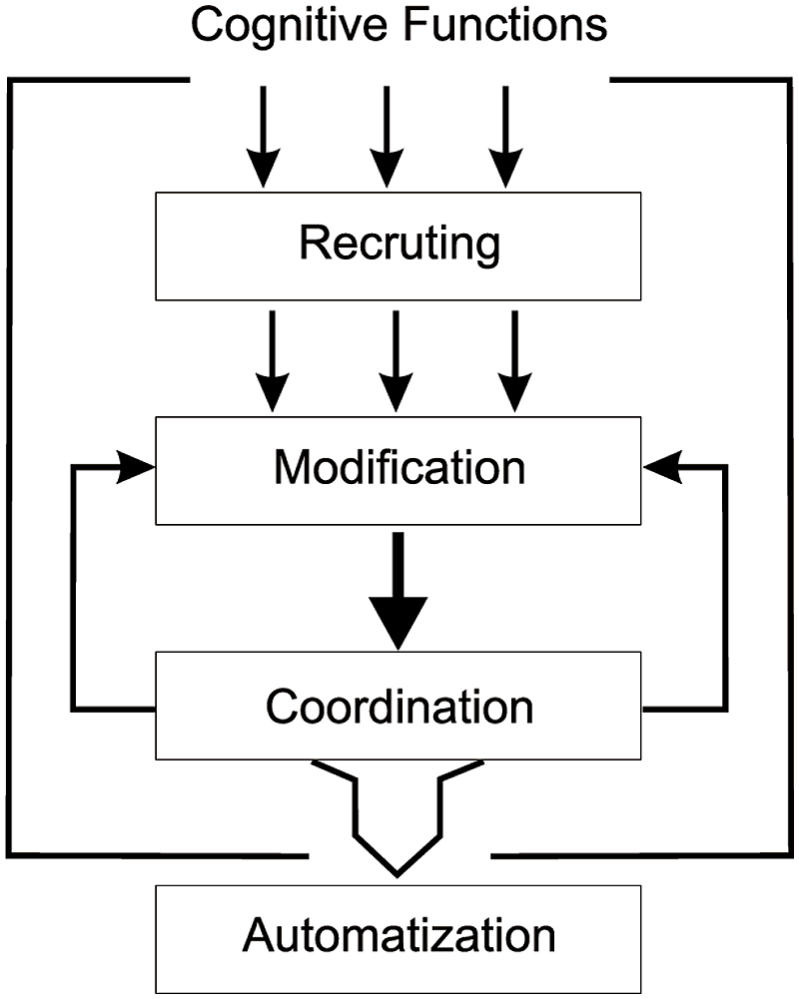
**Functional Coordination Framework for describing the processes involved in learning to read.** Learning to read is described as a form of procedural learning in which, as a consequence of instruction, pre-existing functions and skills, principally from the visual and auditory domain, are recruited, modified and coordinated, leading to cross-modal codes of letters and procedures. After training these get automatized, after which experienced readers are biased against processing strategies for letter perception that do not form part of the procedure. The coordination stage is the most critical one, it stabilizes the modifications. A failure of coordination will result in automatization of an abnormal procedure, leading to reading and writing problems (Lachmann, 2002, 2008). The whole process, including the structural and functional changes related to it, takes several years (Lachmann and van Leeuwen, 2008b; Froyen et al., 2009).

The context-specific process dissociation observed for letters versus non-letters fit a modeling framework (Lachmann, 2002, 2008), schematized in **Figure 5**. The model describes the process of learning to read as a form of procedural learning (Nicolson et al., 2010; Nicolson and Fawcett, 2011) in terms of four stages. We propose that in this process, first, pre-existing skills, principally from the visual and auditory domain, are recruited as a consequence of instruction; for instance, in the perception of script the ability to distinguish small two-dimensional line drawings helps establish letters as the recurring elements of words and sentences. In our interactions with children we scaffold this process by pointing out the distinctive aspects of letters by instantiation, simply like “Look, this is an A,” and by encouraging children to “draw” (rather than write) them. Such abilities are then, in the second stage, modified in a way to optimize their usage in the context of reading and writing, for instance the suppression of orientation invariance and symmetries (“this is not correct, it is upside down”). In other words, this stage involves the emergence of the analytic preference for letters.

Such modifications do not occur in isolation, but co-emerge with the fine-tuning of the phonological system (McBride-Chang, 1999; Lachmann, 2002; Blomert, 2011; Fernandes et al., 2014). These developments take place in a learning context, where both reading and writing are extensively practiced (in fact every day for hours and over years). In this context, considered as third stage in the model, the specific analytic visual abilities and the phonological processing skills become functionally coordinated, giving rise to grapheme-phoneme (reading) and phoneme-grapheme correspondences (writing), leading to cross-modal codes of letters, which form the basis of subsequent automatization processes, the final stage in the model. Given the complexity of these processes, automatization is spread over a period of several years (Lachmann and van Leeuwen, 2008b). Note, that even though children may be able to read and to name letters relatively fast and correctly, i.e., even if they have an established representation of the grapheme-phoneme and the phoneme-grapheme correspondences, the underlying structural und functional basis for its automatization process in the neural system may take 3–4 years (Froyen et al., 2009).

In this framework, developmental dyslexia is not a matter of a deficient automatization per se, but of an automatization of abnormally developed functional coordination (Lachmann, 2002, 2008). Abnormal coordination can be a product of early-stage deficiencies of various kinds: lacking auditory abilities (Ahissar et al., 2000; Talcott and Witton, 2002; Richardson et al., 2004; Goswami, 2011; Groth et al., 2011; Hamalainen et al., 2013), visual instabilities (Slaghuis and Ryan, 1999; Stein and Talcott, 1999; Stein, 2002; Becker et al., 2005) or a combination thereof (Au and Lovegrove, 2007; see Farmer and Klein, 1995, for a review). In these cases, problems may arise already in the recruitment stage; yet they are manifested only in the coordination. This is the case, because the anomalities (e.g., in contrast sensitivity, Slaghuis and Ryan, 1999; or in temporal processing, Steinbrink et al., 2012) at the early levels are not severe enough as to lead to modality-specific deficiencies by themselves. However, such early-stage deficiencies do not *necessarily* lead to problems in coordination, they may be compensated, e.g., by coping strategies or brain plasticity (Frith, 1986).

Alternatively, the anomalies may arise in the “modification” stage, for instance failure to suppress symmetry or other holistic strategies (e.g., von Károlyia et al., 2003; Pegado et al., 2011; Perea et al., 2011) or problems in developing phonological (e.g., Snowling, 2001; Fawcett, 2002) or orthographic skills (Seymour and Evans, 1993). Yet again, even though these problems may arise at this stage, they will be manifested at the coordination level. Failed coordination may lead to compensation strategies resulting in further modifications, just as normal coordination does (see **Figure 5**). For instance, failure to automatically suppress symmetry may lead to active symmetry suppression, which then becomes an engrained strategy. Or, alternatively, it may lead to a strategy of perceiving letters as images just like non-letters (Lachmann and van Leeuwen, 2007).

Functional coordination deficits may arise, however, even without any deficiencies in the recruiting and the modification stage, originating from within the coordination process (Froyen et al., 2011) or resulting from deficiencies in automatization (Nicolson and Fawcett, 2011). Rather than automatization, the coordination level may be most liable to manifest the deficiencies, however, because this is the level where the greatest degree of fine tuning of complex functions is required. Note, that this idea is not inconsistent with the cerebellar approach of Nicolson and Fawcett (2011; Fawcett, 2002) since the cerebellum seems to be essentially involved in such fine tuning and coordination processes (Stoodley and Stein, 2011), including language processing (Ackermann and Hertrich, 2000).

## SUMMERY AND CONCLUSIONS

We discussed evidence relating to the emerging prevalence of analytic processing in the perception of letters, and described its relevance to reading, in the context of a modeling framework for learning to read, the Functional Coordination Model. According to this framework, existing skills are recruited, modified, and coordinated in the process of learning to read. It is not the case, therefore, that new basic skills emerge as a consequence of learning to read; for instance, analytic processing is a resident skill also present in children or non-reading adults (Lachmann et al., 2012). Neither is it the case that reading implies loss of perceptual skills; for instance we are still able to perceive non-letter items analytically or, for that matter, letters holistically, if this is recognized as beneficial to the task (Lachmann and van Leeuwen, 2004; van Leeuwen and Lachmann, 2004). Thus, what has been called “recycling” (Dehaene and Cohen, 2007) of basic perceptual or cognitive abilities does not lead, at least in case of our ability to process visual objects, to any loss of this ability. Rather, what we are looking at is the outcome of procedural learning that has resulted in habits that form the building blocks of complex cognitive skills such as reading.

The question if letters are special, that is, whether they are processed differently as compared to non-letters, may thus be answered affirmatively, but only as long as these are taken as part of a reading process. The habitual tendency to do so is strong enough to be manifest in our experiments, even though these used letters outside of a reading context, as long as the task and presentation conditions are sufficiently similar to those of reading. It is the reading skill as such which is special, not the letter configurations. If we exchange all “a”s in a text by a novel visual symbol and ask our participants to read the text, the novel symbol will be incorporated in the automatized skill rather fast and consequently will be treated as letter. Reading is not a matter of certain letters and sounds, these are only concretizations within a complex, higher-order procedural learning process which takes years to get automatized. Afterward, when perceiving letter stimuli, experienced readers may sometimes experience difficulty in suppressing their modified visual and auditory functions which are part of the automatized coordination. These are then habitually processed as letters, and as a result are special to an experienced reader.

From the point of view that failure in learning to read is the consequence of abnormal coordination followed by the process of automatization, it makes no sense to search for a single cause of reading problems. There might be many possible reasons for failure to become a fluent reader, like those described in different theories of developmental dyslexia (e.g., Farmer and Klein, 1995; Bishop et al., 1999; Snowling, 2001; Fawcett, 2002; Stein, 2002; Ramus et al., 2003; Goswami, 2011). All of these may lead to failures in functional coordination. A consequence of this view is, that isolated training of basic functions, such as visual-auditory integration or temporal processing, may have only limited effects, once automatization is already advanced. In that case the skills must be reorganized and then reautomatized (Klatte et al., 2014).

## Conflict of Interest Statement

The authors declare that the research was conducted in the absence of any commercial or financial relationships that could be construed as a potential conflict of interest.

## Footnotes

1 Note that, theoretically speaking, the dimensions analytic-holistic and whole-part, local-global etc. do not necessarily all refer to the same construct (Wagemans et al., 2012). Here, however, we consider these different aspects simply together as an encompassing visual strategy predominant in object recognition.

2 Recent research (Buetti et al., 2014) suggests that the term “irrelevant” within the context of stimulus-response compatibility effects may be misleading, since, e.g., in flanker tasks, the term “task irrelevant flankers” implies the assumption that distracters are not at all related to the task. This is usually not the case, because they are “attentionally relevant” (Buetti et al., 2014). In the context of our approach, however, in which congruence effects are used to estimate whether the processing strategy is analytic versus holistic, this terminology discussion may be considered *irrelevant*.
